# Sensitization Rates for Various Allergens in Children with Allergic Rhinitis in Qingdao, China

**DOI:** 10.3390/ijerph120910984

**Published:** 2015-09-07

**Authors:** Hang Lin, Rongjun Lin, Na Li

**Affiliations:** 1 Department of Otorhinolaryngology, The Affiliated Hospital of Qingdao University, Qingdao 266003, China; E-Mail: linhang0609@sina.com; 2 Department of Pediatrics, The Affiliated Hospital of Qingdao University, Qingdao 266003, China; E-Mail: linrongjun0312@126.com

**Keywords:** allergic rhinitis, children, allergen, Qingdao, China

## Abstract

The aim of this study was to determine the prevalence of sensitization to common allergens in children with allergic rhinitis (AR) living in Qingdao, China. We conducted a retrospective analysis for AR cases, who underwent skin prick tests (SPT) in Qingdao. A total of 2841 children with AR qualified for the inclusion criteria (Age 3–5 years: 1500 children; Age 6–12 years: 1341 children). The most common inhaled allergens to which the AR children were sensitive were *Dermatophagoides pteronyssinus* (69.3%), *Dermatophagoides farinae* (66.2%) and mould 1 (*Penicillium notatum* 38.9%); while the corresponding ingested allergens were mussel (39.2%), shrimp (36.3%) and carp (36.5%). The prevalence of sensitization to inhaled allergens and food allergens was higher in children >6 years of age as compared to that in children 3–5 years of age (all *p* < 0.05). Children >6 years old were more sensitive to dust mite as compared to children 3–5 years old (*p* < 0.05). Sensitization to dust mite was more common in males than in females (*p* = 0.05). In this study, *Dermatophagoides pteronyssinus* and *Dermatophagoides farinae* were the most common allergens causing AR in children in Qingdao, China. Older children with AR, particularly males, were found to be more sensitive to dust mite.

## 1. Introduction

Allergic rhinitis (AR) is a common allergic disorder involving the respiratory system in both adults and children. It is characterized by inflammation of nasal mucosa caused by an immunoglobulin E (IgE)-mediated immune reaction to specific allergens [1,2]. Clinical manifestations of AR include nasal congestion, nasal itching, nasal catarrh, sneezing, and other allergic symptoms such as ocular pruritis, redness of eyes and/or lacrimation. It seriously affects the study, work efficiency and quality of life. The average overall prevalence of current rhinoconjunctivitis symptoms was 14.6% (range 1.0%–45%) for the 13–14-years old children, and 8.5% for 6–7-years olds [3]. Several recent epidemiological studies have documented an increased incidence of AR in children [4,5,6,7]. Indeed, allergic rhinitis is a global public health problem, and can exert adverse impact on patient’s quality of life. It is also considered to be an independent risk factor for the development of asthma [8,9]. The World Health Organization (WHO) has explicitly defined AR as a global priority, with prevention of AR being an important element of WHO strategy for prevention and treatment of allergic diseases and asthma. Qingdao is a region with a much higher incidence of allergic rhinitis than the inland areas of China. In parallel with the increasing urbanization in Qingdao, the incidence of AR has shown a rising trend. 

Environmental factors play a fundamental role in the causation of AR as well as other respiratory tract allergic disorders [10,11,12]. The prevalence of allergic disorders is closely linked to a variety of environmental allergens and the level of exposure through direct contact, inhalation, or ingestion. Among the wide variety of environmental factors associated with human allergic diseases, dust mite, mould type 1 (*Penicillium notatum*) and type 2 (*Aspergillus fumigatus*), pollen and animal fur are some of the important allergens implicated in AR and asthma [13,14,15]. Clinically, screening for these allergens is very important for diagnosis and administering individualized specific immune therapy. However, the prevalence of sensitization to these allergens varies between different regions and in different population groups. A prospective evaluation of allergens in 175 newly diagnosed AR in children revealed an 85% prevalence of sensitization to traditional dust mites (*Dermatophagoides pteronyssinus* and *Dermatophagoides farinae* mix), and 62% prevalence of sensitization to *Blomia tropicalis*. The overall sensitization rates in this study were 98% for mites, 10% for household pets, 9% for mould type 1 (*Penicillium notatum*) and type 2 (*Aspergillus fumigatus*), and 12% for various food proteins [16]. Studies have suggested dust mites, pollen of summer and autumn, weed pollen, *Neurospora crassa* and *Blattella germanica*as are the leading allergens responsible for causing AR in the northern regions of China [17]. A cross-sectional survey of 6304 patients with asthma and/or rhinitis in 17 cities of China revealed house dust mites as the most prevalent allergens in patients with asthma and/or rhinitis. Further, there was considerable variability in sensitization to the common allergens between different geographical regions as well as inpatients of different ages [18].

Therefore, identification of the specific allergens prevalent in a particular region as well as in various population subsets can facilitate early diagnosis, timely treatment and can help in formulating preventive strategies against respiratory allergic diseases including AR. Although, allergens responsible for AR in children have been reported from a few regions of China [17,18], data on the prevalence of sensitization to common allergens among children in Qingdao region of China remains scarce. The aim of this study was to determine the prevalence of sensitization to allergens in children with AR from Qingdao region of China.

## 2. Methods

### 2.1. Study Subjects

This retrospective study was approved by the Ethics Review Board of Hospital at Qindao, China. A total of 2841 patients with AR, with or without asthma, who received treatment at the departments of Otolaryngology, Head and Neck Surgery and Pediatrics at Qingdao, China, between January 2003 and December 2014, were enrolled in the study. The inclusion criteria were presence of sneezing, or nasal catarrh, itchy or blocked nose in the absence of cold or flu. Written informed consent was obtained from all patients included in the study. Treatment with anti-histaminic drugs and glucocorticoids was discontinued three days and one week prior to the skin prick test (SPT), respectively.

### 2.2. Skin Prick Test (SPT)

Sensitivity to two groups of common allergens (inhaled allergens and ingested allergens) was tested using standard SPT reagent (ALK, Horsholm, Denmark). The test included 10 types of inhaled allergens, including *Dermatophagoides farinae, Dermatophagoides pteronyssinus*, mould type 1 (*Penicillium notatum*), mould type 2 (*Aspergillus fumigatus*), late spring flower, summer autumn flower, weed, ragweed, latex, and Gramineae, respectively. There were 10 ingested allergens, including shrimp, mussel, carp, milk, egg, peanut, peach, eel, hemp, sea crab. Histamine hydrochloride was used as a positive control, while saline was used as a negative control. SPT were performed on the flexor aspect of the forearm. 

Results were recorded 20 min after administering the allergens. The ratio of allergen wheal and histamine wheal was used to evaluate the results. The wheal was measured as the average length of the perpendicular line across its middle point and the longest diameter. A ratio of 0%–25% was considered negative (−); 26%–50% was considered positive (+); 51%–100% was positive (++); 101%–200% was positive (+++); >200% was graded as positive (++++).

### 2.3. Statistical Analysis

All data were analyzed using SPSS version 19.0 software (SPSS Inc., Chicago, IL, USA). The percentage of positive skin test to various allergens was calculated as positive rate (%). Demographic (age, sex) groups and rhinitis group were considered as categorical variables. The differences among variables were evaluated by chi-square test. A two-tailed *p* < 0.05 was considered as statistically significant.

## 3. Results

### 3.1. Baseline Characteristics of Study Population

The study population comprised of 2841 children with AR, with or without asthma (1732 male, 1109 females; average age 6.03 ± 2.81 (mean ± SD) years). There were 1500 children in the age group of 3–5 years (4.24 ± 1.24 years), while, 1341 children were among 6–12 years (9.24 ± 1.02 years) of age.

### 3.2. Leading Allergens Causing AR

The most common inhaled allergens causing AR in children were *Dermatophagoides pteronyssinus* (69.3%), *Dermatophagoides farinae* (66.2%), mould type 1 (*Penicillium notatum*), and mould type 2 (*Aspergillus fumigatus*) (38.9%). The top three leading ingested allergens were mussel (39.2%), shrimp (36.3%) and carp (36.5%). 

**Table 1:** Prevalence of sensitization to allergens in patients by age.

Allergens		Total Patients (N)	%	Age 3–5 years (N)	%	Age > 6 years (N)	%	Chis-Quare (χ^2^)	*p* Value
Inhaled	*Dermatophagoides farinae*	1887	66.4	634	62.5	1253	70.8	22.271	**<0.01**
	*Dermatophagoides pteronyssinus*	1978	69.6	688	67.5	1290	72.0	6.564	**<0.05**
	*Mould type 1 (Penicillium notatum)*	1109	39	386	36.4	723	42.0	9.276	**<0.01**
	*Mould type 2 (Aspergillus fumigatus)*	900	31.7	294	29.3	606	34.3	8.079	**<0.01**
	Late spring flower	1076	37.9	371	36.3	705	39.6	3.206	>0.05
	Summer autumn flower	544	19.1	207	20.6	337	17.5	4.327	**<0.05**
	Weeds	686	24.1	235	23.1	451	25.3	1.781	>0.05
	Ragweed	618	21.8	207	20.3	411	23.4	4.124	**<0.05**
	Latex	690	24.3	217	20.7	473	28.3	21.817	**<0.01**
	Gramineae	712	25.1	228	22.4	484	28.0	11.987	**<0.01**
Ingested	Shrimp	1030	36.3	385	34.5	645	38.6	5.3	**<0.05**
	Mussels	1113	39.2	432	40.0	681	38.7	0.477	>0.05
	Carp	1038	36.5	386	34.7	654	39.0	5.724	**<0.05**
	Milk	708	24.9	270	25.2	438	24.8	0.061	>0.05
	Egg	616	21.7	231	21.5	385	22.1	0.120	>0.05
	Peanut	643	22.6	241	22.4	402	23.1	0.201	>0.05
	Peach	579	20.4	199	20.2	380	20.8	0.188	>0.05
	Eel	198	6.97	94	8.1	104	5.7	6.237	**<0.05**
	Hemp	169	5.95	56	6.5	113	5.4	1.296	>0.05
	Sea crab	43	1.51	12	1.0	31	2.1	5.474	**<0.05**

### 3.3. Comparison of Allergen Sensitizations between Groups with Different Ages

As shown in Table 1, the three most common inhaled allergens causing AR in children from 3–5 years were *Dermatophagoides pteronyssinus* (67.53%), *Dermatophagoides farinae* (62.47%), and mould type 1 (36.4%). Similarly, the three most common inhaled allergens in children from 6–12 years were *Dermatophagoides pteronyssinus* (71.96%), *Dermatophagoides farinae* (70.84%), and mould type 1 (41.98%). The prevalence of sensitization to eight types of inhaled allergens, namely *Dermatophagoides farinae*, *Dermatophagoides pteronyssinus*, mould type 1 and type 2, summer autumn flower, ragweed, latex and gramineae was significantly different between children 3–5 years of age and those >6 years of age (*p* < 0.05). The sensitization rates to *Dermatophagoides farinae* and *Dermatophagoides pteronyssinus* in children >6 years of age were significantly higher than that in 3–5 years old children (χ^2^ = 5.3, *p* < 0.05).

The top three ingested allergens in <6 year age group were mussel (39.60%), carp (34.33%) and shrimp (34.13%). The corresponding allergens in the 6–12 years age group were carp (39%), mussel (38.70%) and shrimp (38.63%). As shown in Table 1, the prevalence of sensitization to shrimp, carp, eel, crab was significantly different between children 3–5 years and those >6 years of age (all *p* < 0.05). 

### 3.4. Comparison of Allergen Sensitization by Sex

The three most common inhaled allergens in males were *Dermatophagoides farinae* (67.6%), *Dermatophagoides pteronyssinus* (70.9%), and mould type 1 and type 2 (73.4%); while *Dermatophagoides pteronyssinus* (67.5%), *Dermatophagoides farinae* (64.4%), and mould type 1 and type 2 (66.2%) were the most common allergens to which females were sensitive. As shown in Table 2, the prevalence of sensitization to mould type 1, ragweed, and latex were found to be significantly different between males and females (all *p* < 0.05). The sensitization rate to both *Dermatophagoides farinae* and *Dermatophagoides pteronyssinus* was significantly higher in males (χ^2^ = 4.3, *p* < 0.05). The prevalence of sensitization to top three digested allergens in male patients was 36.8% for shrimp, 38.8% for mussels, and 36.0% for carp; while in females it was 35.4% for shrimp, 39.8% for mussels, and 37.4% for carp. The prevalence of sensitization to milk in male and female patients showed significant difference (χ^2^ = 17.091, *p* < 0.01). 

**Table 2:** Prevalence of sensitization to allergens by sex.

Allergens		Males (N)	%	Females (N)	%	Chi-Square (χ^2^)	*p* Value
Inhaled	*Dermatophagoides farinae*	1205	67.6	682	64.4	3.088	>0.05
	*Dermatophagoides pteronyssinus*	1263	70.9	715	67.5	3.543	>0.05
	*Mould type 1 (Penicillium notatum)*	724	40.6	385	36.4	5.097	**<0.05**
	*Mould type 2 (Aspergillus fumigatus)*	584	32.8	316	29.8	2.640	>0.05
	Late spring flower	681	18.5	395	37.3	0.237	>0.05
	Summer autumn flower	338	38.2	206	19.5	0.101	>0.05
	Weeds	445	25.0	241	22.8	1.779	>0.05
	Ragweed	410	23.0	208	19.6	4.423	**<0.05**
	Latex	461	25.9	229	21.6	6.511	**<0.05**
	Gramineae	439	24.6	273	25.8	0.463	>0.05
Ingested	Shrimp	655	36.8	375	35.4	0.520	>0.05
	Mussels	692	38.8	421	39.8	0.237	>0.05
	Carp	642	36.0	396	37.4	0.535	>0.05
	Milk	398	22.3	310	29.3	17.091	**<0.01**
	Egg	388	21.8	228	21.5	0.023	>0.05
	Peanut	408	22.9	235	22.2	0.188	>0.05
	Peach	363	20.4	216	20.4	0	>0.05
	Eel	120	6.7	78	9.5	1.788	>0.05
	Hemp	105	5.9	64	6.0	0.027	>0.05
	Sea crab	26	1.5	17	7.4	0.408	>0.05

### 3.5. Comparison of the Intensity of Sensitization to Mite Allergen

**Figure 1:**
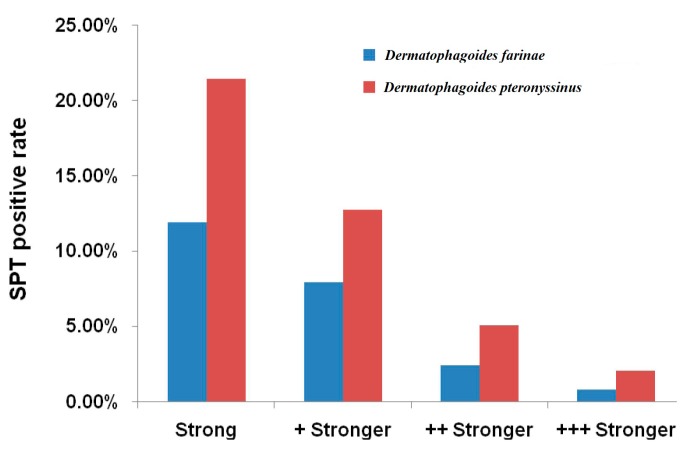
The prevalence and intensity of sensitization to *Dermatophagoides pteronyssinus* and *Dermatophagoides farinae* in patients with allergic rhinitis in Qindao, China from 2003–2014.

The positivity rate in patients allergic to both *Dermatophagoides farinae* and *Dermatophagoides pteronyssinus* was 60.0%. Among these patients, for those sensitive to at least one type of other allergen, the positivity rate was 56.7%. In contrast, the positivity rate was 9.6% in patients sensitive to *Dermatophagoides pteronyssinus* but not *Dermatophagoides farinae*. Among these patients, the positivity rate was 9.4% in patients sensitive to at least one type of other allergens. Whereas, the positivity rate of patients sensitive to *Dermatophagoides farinae* but not to *Dermatophagoides pteronyssinus* was 6.4%. Among these patients, the positivity rate was 5.9% in patients sensitive to at least one other type of allergens. The positivity rate was 21.5% in patients who were not sensitive to both *Dermatophagoides farinae* and *Dermatophagoides pteronyssinus*, but were sensitive to other types of allergens. The positivity rate in patients who had the same intensity of sensitization to both *Dermatophagoides farinae* and *Dermatophagoides pteronyssinus* was 66.63%. The prevalence in patients who had stronger intensity of sensitization to *Dermatophagoides pteronyssinus* than that to *Dermatophagoides farinae* was 21.44%. Among these patients, the positivity rates were 12.81% (+ higher), 5.10% (++ higher), and 2.08% (+++ higher). As shown in Figure 1, when compared with the intensity to *Dermatophagoides farinae*, the intensity of sensitization to dust mite was significantly higher (*p* < 0.05).

## 4. Discussion

In this study, the leading allergens causing AR in Qingdao region were *Dermatophagoides pteronyssinus* (69.6%), *Dermatophagoides farinae* (66.4%), mould type 1 (39%), mussel (39.2%), shrimp (36.3%), and carp (36.5%). The rates of sensitization to inhaled allergens were found to be higher than the sensitization rate to ingested allergens, which suggests that the respiratory allergic diseases are more closely related to inhaled allergens in Qingdao region. Qingdao is located to the south of Shandong Peninsula of China, and abuts the Yellow Sea towards the eastern and the southern region. The region has temperate maritime monsoon climate with high humidity, and is suited to the survival and spread of dust mites and molds. Additionally, the staple diet of the residents in this region includes seafood. Due to the above mentioned factors, dust mite is the most significant inhaled allergen, while mussel and shrimp are the most significant ingested allergens in the region.

Dust mites mainly reside in mattresses, clothing, blankets, carpets, plush toys, and pets such as cats and dogs. Dust mites grow rapidly in dark and damp environments. Each part of the mite body, including its secretions, excretions and shedding are potential allergens. These substances float in the air and are inhaled into the respiratory tract, thus inducing severe allergy [19,20]. According to Xiang *et al.* [21], mildew and indoor smoking were risk factors associated with high levels of dust mites. Studies have shown that the main sources of *Dermatophagoides pteronyssinus* are the bed sheets, pillows, carpets, and towels. The main sources of *Dermatophagoides farinae* include various grains, such as rice and flour [22].

Sensitization to *Dermatophagoides pteronyssinus* is subject to seasonal and regional variations in addition to the species of mites [23]. Studies have shown that the environmental temperature and humidity are the main factors influencing the survival of dust mites. Owing to the different climatic conditions in different regions, the breeding of dust mites differs in different environments. According to Chen, *et al.* [24], mites are the main allergens responsible for pediatric asthma and rhinitis in the Guangzhou region of China. In their study, the prevalence of allergy to *Dermatophagoides pteronyssinus* was as high as 79.8%, followed by *Dermatophagoides farinae* (72.7%) and tropical mites (65.0%). Wu *et al.* [25] reported dust mites as the most common allergen in children belonging to the Chengdu region of China. In their study, 59.49% children were found to be allergic to *Dermatophagoides pteronyssinus*, followed by *Dermatophagoides farinae* (55.26%) and tropical mites (46.31%). In the present study, prevalence of *Dermatophagoides pteronyssinus* was higher than that reported earlier for the Chengdu region; while it was lower than the prevalence reported earlier in the Guangzhou region. Compared with the city of Chengdu, Qingdao is located in the north with temperate maritime monsoon climate, which is more suited to the survival of dust mites. Although both Qingdao and Guangzhou are coastal cities, the relatively higher volume of city traffic and the resultant air pollution provides a more conducive environment for survival of dust mites in the Guangzhou region. Various studies have shown that limiting the exposure of sensitive children to dust mites can significantly control allergic symptoms. A study involving 160 cases of asthmatic children showed significant improvement in asthmatic symptoms after dust mite intervention as compared to that without dust mite intervention [26]. With the dust mite environmental intervention, the prevalence of severe asthma reduced from 45% to 22%.

People are usually allergic to many allergens simultaneously. The synergistic effects may aggravate the allergic symptoms, and often reduce the efficacy of single allergen immunotherapy. In the present study, allergy to only a single allergen was found to be rare. The majority of children were sensitive to >1 allergens, and especially to both dust mite and *Dermatophagoides pteronyssinus*, with rates as high as 60%. Among these patients, 56.7% were allergic to one or more types of other allergens. The intensity of sensitization to *Dermatophagoides farinae* was significantly higher than that to *Dermatophagoides pteronyssinus*. Saridomichelakis, *et al.* [27] reported a high degree of cross reactivity between *Dermatophagoides farinae* and *Dermatophagoides pteronyssinus* in animals. It is unclear whether the allergy to both types of mites occurs simultaneously or there are similar allergens in both types of mites. Currently, clinical desensitization therapies mainly target a single allergen, *i.e.*, against dust mite or *Dermatophagoides pteronyssinus*. It remains unclear if this treatment is also effective against other type of mites.

In the present study, we found children >6 years of age were significantly more sensitive to three types of inhaled allergens, including *Dermatophagoides pteronyssinus* (67.53%), *Dermatophagoides farinae* (62.47%), and mould type 1 (36.4%), when compared with children 3–5 years of age. Similarly, epidemiological studies in Beijing and Guangzhou regions showed that *Dermatophagoides pteronyssinus*, dust mites, and cat hair are major risk factors causing allergy in children aged 9–11 years with asthma and bronchial hyperresponsiveness [28]. Another study in Guangzhou area showed a significantly higher prevalence of allergy to mites, cat hair/fur, and cockroaches in children with respiratory allergic diseases who were >7 years of age [24]. As age increases, older children (6–12 years) with AR, especially males, become more sensitive to dust mites. It is likely due to the increased locomotor activity in older children that increases the exposure to inhaled allergens.

In this study, children with allergic rhinitis were mainly sensitive to three types of food allergens, *i.e.*, mussel, shrimp and carp. It suggests that ingested allergens also play an important role in the occurrence of allergic airway diseases. Therefore, it is recommended that children with allergic asthma and AR should be careful when consuming seafood. In addition, the sensitivity to ragweed and pollen was found to be much higher in children with allergic rhinitis. It is likely due to the expansion of the green environment and the import of foreign plant species (such as Ambrosia) in the Qingdao region. Skin Prick Test is a commonly used diagnostic modality for identifying a specific allergen(s) to which an individual is sensitive. It is simple, fast, safe and highly specific [29,30]. Its accuracy is ≥95%, though the test results can potentially be influenced by various factors, such as sample reagent, medication and the allergen type [31]. In the present study, all 2841 patients underwent SPT, and none of them developed adverse reactions.

## 5. Conclusions

*Dermatophagoides farinae* and *Dermatophagoides pteronyssinus* are the most common allergens causing AR in children in Qingdao, China. The sensitization to both allergens usually occurs at the same time. It is often accompanied by sensitization to one or more of the other types of allergens. With the increase in age, children with AR, especially males, become more sensitive to dust mites. Therefore, children with respiratory allergic diseases should avoid or reduce exposure to various allergens, especially dust mites. The specific desensitization treatment available against certain allergens can also be employed for treatment of respiratory allergic diseases in children.
